# Evaluation of synthetic peptides derived from glycoproteins gp45 and gp90 as candidates for the immunodiagnosis of equine infectious anemia

**DOI:** 10.1007/s11259-026-11222-3

**Published:** 2026-05-07

**Authors:** Aníbal Domínguez-Odio, Laura Iliana Coroás González, Dayamí Martín Alfonso, Ernesto Rodríguez Martínez, Miguel Ángel Bedoya Ríos, Daniel Leonardo Cala-Delgado

**Affiliations:** 1 Dirección de Ciencia e Innovación. Grupo Empresarial LABIOFAM, Avenida Independencia km 16 ½, La Habana, Cuba; 2 Centro de Investigaciones Científicas de la Defensa Civil, Carretera de Tapaste y Autopista Nacional Km 23 ½, San José de las Lajas, Cuba; 3 Instituto de Cibernética, Matemática y Física. Calle 15 # 551 e/ C y D, La Habana, Cuba; 4 Grupo de Investigación en Ciencia Animal, Facultad de Medicina Veterinaria y Zootecnia, Universidad Cooperativa de Colombia, sede Bucaramanga. Carrera 33 N°. 30ª-05 (4.162,49 km), Bucaramanga, 68000 Colombia

**Keywords:** Equine infectious anemia, Diagnosis, Synthetic peptide, ELISA

## Abstract

The international scientific community continues to promote the development of diagnostic techniques with improved performance and novel capture antigens. On this basis, two synthetic peptides derived from gp45 and gp90 were evaluated to identify the best candidate for the indirect immunodetection of infection. In both assays, 68 serum samples from naturally infected horses in Granma Province (59 seropositive and 9 seronegative), previously classified using the agar gel immunodiffusion (AGID) test, were analyzed. The diagnostic performance of each candidate peptide was assessed by calculating sensitivity, specificity, positive predictive value, negative predictive value, and the Kappa coefficient. Compared with ELISA_P15/gp90_ and the AGID test, the ELISA_P05/gp45_ variant showed better performance, accurately identifying the highest number of infected animals (91.2%, 56/59). Consequently, it achieved high values for positive predictive value (98.2%), overall accuracy (94.1%), and Kappa coefficient (0.96; very good). The ability of synthetic peptide P05 to discriminate between infected and uninfected animals exceeded that of P15. Based on these results, synthetic peptide P05 may serve as an alternative capture antigen for evaluating the immune response in equine infectious anemia.

## Introduction

The development of immunodiagnostic tools is one of the most urgent challenges currently faced by veterinary medicine. Identifying the presence of pathogens by detecting either antibodies or antigens is essential for controlling several veterinary infectious diseases. Therefore, research in this field has global relevance and direct applications in health surveillance (Aguilar-Montes de Oca et al. 2022). In this context, pathogens with global distribution attract considerable scientific interest because they cause significant economic losses due to persistent infections or multisystemic diseases, in addition to the absence of effective vaccines or treatments.

In this regard, the equine infectious anemia (EIA) virus—classified within the family *Retroviridae*, subfamily *Orthoretrovirinae*, and belonging to the genus *Equine lentivirus*—is a clear example (Simmonds et al. 2024; Tiago et al. 2022). Notification of its etiological agent is mandatory for all countries that are members of the World Organization for Animal Health (WOAH), requiring trade restrictions, selective culling of diseased animals, or permanent segregation (Alnaeem and Hemida 2019; Machado et al. 2021). Global efforts to control this virus are challenged by several factors, including the absence of preventive vaccines, genetic variability among strains (Dantas et al. 2020), the rapid virus evolution resulting from its high replication rate, the poor error-correcting capacity of its polymerase (Dorey-Robinson et al. 2019), and limited knowledge of its molecular epidemiology (Jara et al. 2020).

Occasionally, the official method (immunoprecipitation/Coggins technique) may yield inconsistent or challenging results. These inconsistencies are associated with non-specific reactions caused by low antibody levels in infected animals (Scicluna et al. 2013). The low sensitivity reported by several international authors, along with the subjective judgment of the analyst when interpreting results (Bannai et al. 2023; Issel et al. 2013a, b), among other practical drawbacks, has stimulated research and development of novel diagnostic procedures. In this regard, international efforts aim to detect nucleic acids (Li et al. 2023), develop rapid tests (Zhang et al. 2024), and combine different serological tests (Scicluna et al. 2019).

To achieve these objectives, new molecules are being investigated to facilitate, improve, accelerate, and reduce the cost of detecting positive cases of the disease (Russi et al. 2023), starting from known structures such as the transmembrane glycoprotein gp45 and the surface glycoprotein gp90 (Du et al. 2018; Reis et al. 2012). Scientific and commercial interest in these two proteins arises from their role in viral attachment to the cytoplasmic membrane of host cells (monocytes and macrophages) and from their function as sites of contact between infected and healthy cells, thereby facilitating pathogen entry into susceptible hosts and the progression of infection (Cook et al. 2013). Considering the above, two synthetic peptides derived from gp45 and gp90 were evaluated to identify the best candidate for the development of an indirect immunodetection system for specific antibodies against EIA.

## Materials and methods

### Synthetic peptides

The search for novel candidate antigens for detecting specific antibodies against EIA involved predicting the antigenicity of selected regions of gp45 and gp90 encoded by the env gene using the DNA Star tool (Madison, Wisconsin, USA). In this study, two candidate peptide sequences were identified: P05 and P15 (patent pending). These sequences have not been previously reported in the scientific literature (Naves et al. 2019; Ostuni et al. 2023; Soutullo et al. 2001; Soutullo et al. 2007).

Both peptides were derived from a wild pathogenic strain circulating in Cuba, whose partial sequence is available in GenBank under accession number HQ853234.1 (Díaz-Miranda et al. 2012). The first peptide has a folded structure comprising 26 amino acids and originates from the transmembrane glycoprotein gp45. It is partially located between amino acid residues 511 and 557 within the major immunodominant domain, sharing 84% homology with the Wyoming reference strain. The second peptide comprises 25 amino acids. It is derived from the surface glycoprotein gp90, has a linear structure, is located near the carboxy-terminal end of the R12 region, and shares 75% homology with the Wyoming reference strain.

The two candidate synthetic peptides were obtained at the Center for Genetic Engineering and Biotechnology (Cuba) by solid-phase chemical synthesis using the Boc strategy in polypropylene bags (Hernández et al. 2009). Each 30 × 40 mm bag contained 60 mg of polystyrene polymer resin with 1% divinylbenzene as the solid support. The amino acid corresponding to the C-terminus of the peptide was coupled to the solid support, and the remaining amino acids were gradually assembled in repeated cycles of coupling and Nα-amino group deprotection until the desired peptide sequence was completed. At the end of assembly, the side chains were deprotected, and the peptides were cleaved from the support according to Amblard et al. (2006).

The synthetic peptides were purified by reversed-phase high-performance liquid chromatography (Pharmacia, LKB). An RP-C18 column (Vydac; 25 × 250 mm, 25 μm) was used with a linear gradient from 0% to 60% solution B for 35 min at a flow rate of 5 mL/min. The mobile phase consisted of solution A (0.1% trifluoroacetic acid (TFA) and 2% acetonitrile in H_2_O) and solution B (0.05% TFA in acetonitrile) (Hernández et al. 2001). Chromatograms were recorded at 226 nm, and data were processed using Unicom version 4.11 (GE Healthcare, USA) and Biocrom (CIGB, Cuba) software, yielding a purity of ≥ 95%. The purified peptides were subsequently frozen at − 70 °C for 4 h and lyophilized in a freeze dryer (LABCONCO, USA) for 48 h.

### Research samples

A total of 68 equine serum samples (59 positive and 9 negative), previously classified using the AGID technique at the Central Unit of Agricultural Health Laboratories of Cuba, were used. The clinical samples originated from the province of Granma, the area with the highest incidence and prevalence of EIA in Cuba, and were collected from working and draft crossbred adult horses (8.2 ± 2.4 years old), including 30 males and 38 females. The number of sera included in the panel was determined based on similar international quantitative, descriptive, and comparative studies that employed enzyme-linked immunosorbent assays to diagnose EIA (Cabrera et al. 2025; Nardini et al. 2017; Niu et al. 2024). All sera were stored at 2–8 °C until use. No experimental infections were performed.

### Enzyme-linked immunosorbent assay

Prior to this study (results not shown), preliminary evaluations were conducted to identify the effects of synthetic antigen concentration (2.5 µg/mL, 5 µg/mL, and 10 µg/mL), temperature (4 °C and 37 °C), and incubation time (2 h and 20 h) on the coating of MaxiSorp polystyrene microplates (Thermo Fisher Scientific, USA). Once these variables had been optimized, a concentration of 2.5 µg/mL was selected for each candidate peptide, diluted in sodium bicarbonate buffer (pH 9.6). The coating solution (100 µL per well) was dispensed, and the plates were incubated for 2 h at 37 °C.

Each microplate was then washed twice with a balanced solution containing sodium dihydrogen phosphate (0.6 g/L), sodium chloride (4 g/L), potassium chloride (0.2 g/L), and sodium hydrogen phosphate dodecahydrate (1.9 g/L), adjusted to pH 7.2–7.4 and supplemented with 0.31% Tween 20. Each candidate (P05/_gp45_ and P15/_gp90_) was blocked with a 0.2% solution of bovine serum albumin (fraction V, Sigma Aldrich, USA) and dried at room temperature for 24 h before being sealed in polylaminated aluminum bags (Polylabo, France). Plates were stored for 15 days at 2–8 °C until use.

For each assay, 5 µL of serum and 95 µL of diluent (PBS, pH 7.3) were added per well. The microplates were gently shaken to ensure proper homogenization and incubated for 30 min at 37 °C ± 2 °C. The wells were washed six times with 300 µL of washing solution containing sodium chloride (100 g/L), sodium dihydrogen phosphate (15 g/L), potassium chloride (5 g/L), and sodium hydrogen phosphate dodecahydrate (47.5 g/L), supplemented with 1.25% Tween 20 (Merck, Germany) and adjusted to pH 6.0–6.2. The plates were blotted dry using absorbent paper. Then, 100 µL of conjugate solution (sheep anti-equine IgG conjugated to horseradish peroxidase, diluted in its corresponding buffer) was added. The microplates were incubated for 1 h at 37 °C ± 2 °C in a humid chamber, washed four times with a wash solution, and dried. Next, 100 µL of tetramethylbenzidine substrate (Sigma-Aldrich, USA) was added to each well, and the microplates were incubated in the dark at room temperature for 15 min. The reaction was stopped with 100 µL of 2 M sulfuric acid.

Data analysis was performed using the cutoff-value method (Ochoa 2013). Serum samples (*n* = 68) were tested in 12 replicates for each enzyme-linked immunosorbent assay (ELISA) variant (ELISA_P05/gp45_ and ELISA_P15/gp90_). Each plate was read three times at an absorbance of 492 nm, and results were expressed as the mean. The ratio (XPS/XNS) was then calculated, where XPS represents the mean absorbance of positive serum and XNS represents that of negative serum. A preliminary cutoff value was established based on the formation of a clear upper plateau in optical density readings for positive sera and a lower plateau for negative sera.

### AGID test

The AGID test was performed according to the manufacturer’s instructions (LABIOFAM, Cuba). Petri dishes (90 × 15 mm) containing 15 mL of 1% agar were used. The solidified agar was punched using a mold creating one central well and six peripheral wells. Each well was 5.3 mm in diameter and spaced 2.4 mm apart. The p26 antigen was added to the central well. The peripheral wells were alternately filled with positive control serum and diagnostic target serum samples (24 µL each). AGID results were interpreted as positive if a curved precipitation line was observed and negative if no line appeared after 48–72 h of incubation at 20–25 °C. Tests were considered valid only if the positive and negative controls on each plate yielded the expected results. Samples with uncertain results were reanalyzed (Bannai et al. 2023).

### Data analysis

After serological evaluation at each stage, sensitivity, specificity, positive predictive value, negative predictive value, overall accuracy, and Kappa coefficient were calculated according to Ochoa et al. (2000). Agreement levels based on Kappa values were interpreted as follows: poor (< 0.20), regular (0.21–0.40), moderate (0.41–0.60), good (0.61–0.80), and very good (0.81–1.00). In addition, McNemar’s nonparametric chi-square test was used to detect significant differences between the top-performing tests and the AGID reference method.

## Results

Table 1 summarizes the diagnostic performance of the two synthetic peptide candidates for detecting antibodies against EIA, benchmarked against the AGID test. In general terms, the ELISA_P05/gp45_ variant was shown to have better performance than ELISA_P15/gp90_ in the serum panel. A detailed analysis confirmed that the first candidate accurately detected the highest number of infected animals (sensitivity: 94.9%, 56/59). Furthermore, ELISA_P05/gp45_ demonstrated near-optimal diagnostic efficacy—defined as the overall accuracy in correctly classifying both positive and negative samples—of 94.1%, compared with 89.7% for ELISA_P15/gp90_.

**Table 1:** Distribution of diagnostic results by assay variants (ELISA_P05/gp45_ and ELISA_P15/gp90_)

ELISA_P05/gp45_	AGID AIE, Cuba			Diagnostic performance indicators
	Positive	Negative	Total	
Reactive	56	1	57	Sensitivity: 94.9%
				Specificity: 88.8%
No reactive	3	8	11	Positive predictive value: 98.2%
				Negative predictive value: 72.7%
Total	59	9	**68**	Efficacy: 94.1%
				Kappa coefficient: 0.96 (very good)
ELISA_P15/gp90_				
Reactive	53	1	54	Sensitivity: 89.8%
				Specificity: 88.9%
No reactive	6	8	14	Positive predictive value: 98.1%
				Negative predictive value: 57.1%
Total	59	9	**68**	Efficacy: 89.7%
				Kappa coefficient: 0.94 (very good)

**Table 2:** Differences in diagnostic performance between the best variants (ELISA_P05/gp45_ and ELISA_P15/gp90_) and the reference test (AGID) for AIE

ELISA variants	*n*	Statistic	d.f.	Significance (*P* value)
ELISA _P05/gp45_	68	4.2250	1	0.0398*
ELISA _P15/gp90_	68	4.8913	1	0.0270

*Significance when applying McNemar comparison test

## Discussion

The development of diagnostic techniques aimed at improving control measures and achieving eradication of the EIA virus has been the focus of numerous international studies (Ricotti et al. 2016). Regardless of the capture molecule used, these efforts share the common goal of achieving a reliable diagnostic method capable of identifying infection at an early stage, thereby limiting viral spread among susceptible populations (Espasandin et al. 2021; Li et al. 2023; Zhang et al. 2024).

Among the available diagnostic approaches, indirect ELISAs based on synthetic peptides stand out because of their ability to accurately recognize specific antigen–antibody binding regions (Aguilar-Montes de Oca et al. 2022; Ostuni et al. 2023; Russi et al. 2023) and their cost-effectiveness (Naves et al. 2019 ]. In this study, the selected synthetic peptide (P05) accurately detected both infected and uninfected animals, suggesting its potential application in commercial ELISAs for the indirect immunodiagnosis of EIA (Du et al. 2018; Pandey et al. 2021; Thakur et al. 2022). In this context, it is important to note that the use of the novel peptide is not intended to replace the AGID test as a confirmatory or official test, but to complement the diagnosis as a screening tool.

The synthetic peptides may have limitations related to their structural conformation (linear or folded) and their relatively weak binding to microtiter plate surfaces. The international literature indicates that linear sequences, unlike folded ones, have limited capacity to remain immobilized in the solid phase and effectively recognize target proteins (Soutullo et al. 2007). This may affect diagnostic performance, particularly during early EIA infections when antibody concentrations are low, making it challenging to accurately identify positive animals (Aguilar et al. 2013; Ricotti et al. 2016). These factors as a whole may be present in the peptide constructs studied (P05 and P15), and thus could explain the high diagnostic yield observed in the former and the low yield in the latter.

Based on the ELISA_P05/gp45_ results, the use of a specific gp45-derived peptide sequence as antigen demonstrated acceptable specificity and sensitivity for the system under development. In terms of sensitivity (94.9%), the observed results were superior to those obtained by other ELISAs using recombinant gp45 alone (90.0%) (Du et al. 2018), p26 antigens (90.4%) (Villa-Mancera et al. 2024), and gp90 protein antigens (91.11%) (Santos da Silva et al. 2022) as capture antigens. However, the values obtained in this study were lower than those reported for ELISAs involving synthetic gp45 peptides (Naves et al. 2019), gp45/p26 combinations (Ostuni et al. 2023), and gp90/45 antigen mixtures (Russi et al. 2023), which achieved sensitivity levels of 98.6%, 96.0%, and 99.5%, respectively.

Comparative analysis shows that the P05 peptide readily detects anti-EIA antibodies and remains a viable option for identifying antibodies with minimal interference risk (Issel et al. 2013a, b; Santos da Silva et al. 2022). It also presents higher diagnostic sensitivity compared with the AGID test (Santos da Silva et al. 2022), a desirable feature for large-scale EIA diagnostics (Machado et al. 2021; Vieira et al. 2023). Nonetheless, these results require validation using additional clinical samples to confirm their functional characteristics. Further studies should also assess its diagnostic performance relative to currently available commercial tests. A key limitation to address in the immediate future is the need to increase the number of clinical samples processed, as recommended by comparable international studies (Acevedo-Jiménez et al. 2026; Russi et al. 2023; Naves et al. 2019). This step is essential for the ELISA evaluation to be useful and to reflect its true diagnostic performance (Table 2).

## Conclusion

Under the conditions tested, the synthetic peptide variant P05 demonstrated the best diagnostic performance for serological detection of anti‑EIA antibodies.

## Data Availability

No datasets were generated or analysed during the current study.

## References

[CR1] Acevedo-Jiménez G, Morales-González C, Akbarin M, Rodríguez-Murillo C, González-Fernández V, García-Flores M, Alvarez H (2026) Synthetic gp90 peptide ELISA for Equine Infectious Anemia virus: improved sensitivity and risk factor insights. Prev Vet Med 106811. 10.1016/j.prevetmed.2026.106811 10.1016/j.prevetmed.2026.106811 41719807

[CR2] Aguilar A, Camacho F, Amin N, Prieto J, Garay H, Reyes O (2013) Comparación de la antigenicidad de dos construcciones peptídicas de mimotopos del virus de la hepatitis A mediante suero de ratones inmunizados. Vacumonitor 223:43–46

[CR3] Aguilar-Montes de Oca S, Montes-de-Oca J, Vázquez-Chagoyán C, Barbabosa-Pliego A, Rivadeneira-Barreiro P, Zambrano-Rodríguez P (2022) El uso de péptidos en el serodiagnóstico veterinario de enfermedades infecciosas: una revisión. Vet Sci 9:561. 10.3390/vetsci9100561 36288174 10.3390/vetsci9100561 PMC9610506

[CR4] Alnaeem A, Hemida M (2019) Vigilancia del virus de la anemia infecciosa equina en el este y centro de Arabia Saudita durante 2014–2016. Vet World 12(5):719–723. 10.14202/vetworld.2019.719-723 31327910 10.14202/vetworld.2019.719-723 PMC6584864

[CR5] Amblard M, Fehrentz JA, Martinez J, Subra G (2006) Métodos y protocolos de síntesis moderna de péptidos en fase sólida. Mol Biotechnol 33:239–254. 10.1385/MB:33:3:239 16946453 10.1385/MB:33:3:239

[CR6] Bannai H, Kambayashi Y, Nemoto M, Yamanaka T, Tsujimura K (2023) Comparación de 4 kits de inmunodifusión en gel de agar para la detección serológica de anticuerpos contra el virus de la anemia infecciosa equina. J Vet Diagn Invest 35(4):430–432. 10.1177/10406387231171567 37129383 10.1177/10406387231171567 PMC10331386

[CR7] Cabrera S, Vanegas B, Durán T, Fiallos C, Cabrera T, Pico Z (2025) Precisión diagnóstica y eficiencia operativa de la inmunodifusión en gel de agar-agid, frente al ELISA en la vigilancia epidemiológica de la anemia infecciosa equina. Rev Cient Mult 5:6453–6466. 10.37811/cl_rcm.v9i5.20004

[CR8] Cook RF, Leroux C, Issel CJ (2013) Anemia infecciosa equina y virus de la anemia infecciosa equina en 2013: una revisión. Vet Microbiol 167(1–2):181–204. 10.1016/j.vetmic.2013.09.031 24183747 10.1016/j.vetmic.2013.09.031

[CR9] Dantas M, Gorzoni F, Ferreira C, Magro A, Geessien K, Moura de Aguiar D (2020) Alta variabilidad genómica del virus de la anemia infecciosa equina obtenido de caballos infectados naturalmente en el Pantanal, Brasil: un caso endémico. Viruses 85:102852. 10.3390/v12020207

[CR10] Díaz-Miranda M, Vázquez-Blomquist D, Cruz L, Vasallo C, Campos T, Pérez J, Duarte C (2012) Secuencia del gen que codifica la proteína p26 de una cepa cubana del Virus de la Anemia Infecciosa Equina. Biotec Aplic 29(1):17–21

[CR11] Dorey-Robinson D, Locker N, Steinbach F, Choudhury B (2019) Caracterización molecular de las cepas del virus de la anemia infecciosa equina detectadas en Inglaterra en 2010 y 2012. Transbound Emerg Dis 66(6):2311–2317. 10.1111/tbed.13286 31267701 10.1111/tbed.13286

[CR12] Du C, Hu Z, Hu S, Lin Y, Wang X, Li Y (2018) Desarrollo y aplicación de una prueba ELISA indirecta para la detección de anticuerpos gp45 contra el virus de la anemia infecciosa equina. J Equine Vet Sci 62:76–80. 10.1016/j.jevs.2017.10.018

[CR13] Espasandin A, Cipolini M, Forletti A, Díaz S, Soto J, Martínez D, Storani C, Monzón N, Beltrame J, Lucchesi E, Soto P (2021) Comparación de técnicas serológicas para el diagnóstico de anemia infecciosa equina en una zona endémica de Argentina. Métodos J Virol 291:114101. 10.1016/j.jviromet.2021.114101 10.1016/j.jviromet.2021.114101 33609629

[CR14] Fuente-Alba C, Molina V (2017) Razón de verosimilitud: definición y aplicación en Radiología. Rev Argent Radiol 81:204–220

[CR15] Hernández M, Pozo L, Amat M, Rodríguez C, Higginson D (2009) Comparación de la antigenicidad de un péptido sintético y una proteína recombinante de la región detransmembrana (gp 36) del VIH-2. Revista CENIC Cien Biol 40(1):59–62

[CR16] Hernández M, Pozo L, Gómez I, Melchor A (2001) Antigenicidad de dos péptidos sintéticos de la región de transmembrana (gp4l) del VIH-1 y su utilidad en el inmunodiagnóstico. Revista CENIC Cien Biol 32(2):113–117

[CR18] Issel CJ, Scicluna MT, Cook SJ, Cook RF, Caprioli A, Ricci I, Rosone F, Craigo JK, Montelaro RC, Autorino GL (2013b) Challenges and proposed solutions for more accurate serological diagnosis of equine infectious anaemia. Vet Rec 172(8):210. 10.1136/vr-2012-100735 23161812 10.1136/vr-2012-100735 PMC3593188

[CR17] Issel C, Scicluna M, Cook S, Cook R, Caprioli A, Ricci I (2013a) Desafíos y soluciones propuestas para un diagnóstico serológico más preciso de la anemia infecciosa equina. Vet Rec 172(8):210. 10.1136/vr-2012-100735 23161812 10.1136/vr-2012-100735 PMC3593188

[CR19] Jara M, Frias-De-Diego A, Machado G (2020) Filogeografía del virus de la anemia infecciosa equina. Frente Ecol Evol 8:127. 10.3389/fevo.2020.00127

[CR20] Li S, Guo K, Wang X, Lin Y, Wang J, Wang Y, Du C, Hu Z, Wang X (2023) Desarrollo y evaluación de una PCR cuantitativa en tiempo real para la detección del virus de la anemia infecciosa equina. Microbiol Spectr 11(6):e0259923. 10.1128/spectrum.02599-23 37811976 10.1128/spectrum.02599-23 PMC10715080

[CR21] Machado G, Corbellini L, Frias-De-Diego A, Dieh G, Dos Santos D, Jara M, de Freitas C (2021) Impacto de los cambios en las regulaciones de movimiento de los caballos sobre los riesgos de anemia infecciosa equina: un enfoque de evaluación de riesgos. Anterior Vet Med 190:105319. 10.1016/j.prevetmed

[CR22] Nardini R, Autorino GL, Issel CJ, Cook RF, Ricci I, Frontoso R, Rosone F, Scicluna MT (2017) Evaluation of six serological ELISA kits available in Italy as screening tests for equine infectious anaemia surveillance. BMC Vet Res 13:105. 10.1186/s12917-017-1007-6 28410613 10.1186/s12917-017-1007-6 PMC5391595

[CR23] naves J, Oliveira F, Bicalho J, Santos P, Machado-de-Ávila R, Chavez-Olortegui C (2019) Diagnóstico serológico de anemia infecciosa equina en caballos, burros y mulas mediante ELISA con un péptido sintético gp45 como antígeno. Métodos J Virol 266:49–57. 10.1016/j.jviromet.2018.12.009

[CR24] Niu X, Liu Q, Wang P, Zhang G, Jiang L, Zhang S, Zeng J, Yu Y, Wang Y, Li Y (2024) Establishment of an indirect ELISA method for the detection of the bovine rotavirus vp6 protein. Animals 14:271. 10.3390/ani14020271 38254440 10.3390/ani14020271 PMC10812791

[CR25] Ochoa R (2013) Técnicas inmunoenzimáticas en el desarrollo clínico de vacunas. Ediciones Finalay, La Habana, Cuba

[CR26] Ochoa R, Martínez JC, Ferriol X, Estrada E, García AM, Blanco R, Sotolongo F (2000) Guía para la estandarización de técnicas inmunoenzimáticas en ensayos de vacunas. Vaccimonitor 9(3):13–18

[CR27] Ostuni A, Iovane V, Monné M, Crudele MA, Scicluna M, Nardini R, Raimondi P, Frontoso R, Boni R, Bavoso A (2023) Un antígeno polipéptido TM de doble cepa (gp45) y su aplicación en el serodiagnóstico de la anemia infecciosa equina. Métodos J Virol 315:114704. 10.1016/j.jviromet.2023.114704 10.1016/j.jviromet.2023.114704 36842487

[CR28] Pandey S, Malviya G, Chottova Dvorakova M (2021) Rol de los péptidos en el diagnóstico. Int J Mol Sci 22(16):8828. 10.3390/ijms22168828 34445532 10.3390/ijms22168828 PMC8396325

[CR29] Reis JK, Diniz RS, Haddad JP, Ferraz IB, Carvalho AF, Kroon EG, Ferreira PC, Leite RC (2012) La prueba ELISA de proteína de envoltura recombinante (rgp90) para el virus de la anemia infecciosa equina proporciona resultados comparables a los de la inmunodifusión en gel de agar. J Virol Methods 180:62–67. 10.1016/j.jviromet.2011.12.012 22227617 10.1016/j.jviromet.2011.12.012

[CR30] Ricotti S, Garcia MI, Veaute C (2016) Infecciones serológicamente asintomáticas y ocultas por el virus de la anemia infecciosa equina (VAIE) en caballos. Vet Microbiol 187:41–49. 10.1016/j.vetmic.2016.03.007 27066707 10.1016/j.vetmic.2016.03.007

[CR31] Russi R, Garcia L, Cámara M, Soutullo A (2023) Validation of an indirect in-house ELISA using synthetic peptides to detect antibodies anti-gp90 and gp45 of the equine infectious anaemia virus. Equine Vet J 55(1):111–121. 10.1111/evj.13555 35007356 10.1111/evj.13555

[CR32] Scicluna M, Autorino G, Cook S, Issel C, Cook R, Nardini R (2019) Validación de un ensayo de inmunotransferencia con un sistema de lectura objetivo, utilizado como prueba confirmatoria en programas de vigilancia de anemia infecciosa equina. J Virol Methods 266:77–88. 10.1016/j.jviromet.2019.01.012 30684508 10.1016/j.jviromet.2019.01.012

[CR33] Scicluna M, Issel C, Cook F, Manna G, Cersini A, Rosone F (2013) ¿Es adecuado un sistema de diagnóstico basado exclusivamente en inmunodifusión en gel de agar para controlar la propagación de la anemia infecciosa equina? Vet Microbiol 165(1–2):123–134. 10.1016/j.vetmic.2013.02.027 23618837 10.1016/j.vetmic.2013.02.027

[CR34] Simmonds P, Adriaenssens EM, Lefkowitz EJ (2024) Cambios en la taxonomía de virus y los Estatutos del ICTV ratificados por el Comité Internacional de Taxonomía de Virus. Arch Virol 169(11):236. 10.1007/s00705-024-06143-y 39488803 10.1007/s00705-024-06143-y PMC11532311

[CR35] Soutullo A, Santi M, Perin J, Beltramini L, Borel I, Frank R, Tonarelli G (2007) Análisis sistemático de epítopos de la proteína central p26 EIAV. J Mol Recognit 20(4):227–237. 10.1002/jmr.825 17705340 10.1002/jmr.825

[CR36] Soutullo A, Verwimp V, Riveros M, Pauli R, Tonarelli G (2001) Diseño y validación de una prueba ELISA para el diagnóstico de anemia infecciosa equina (AIE) mediante péptidos sintéticos. Vet Microbiol 79(2):111–121. 10.1016/s0378-1135(00)00352-7 11230933 10.1016/s0378-1135(00)00352-7

[CR37] Thakur R, Suri CR, Kaur I, Rishi P (2022) Péptidos como herramientas diagnósticas, terapéuticas y teranósticas: avances y retos futuros. Crit Rev Ther Drug Carrier Syst 40(1):49–100. 10.1615/CritRevTherDrugCarrierSyst.2022040322 36374841 10.1615/CritRevTherDrugCarrierSyst.2022040322

[CR38] Tiago S, Ribas P, Benevides S, Silva S, Moreira O, Barbosa C (2022) Aspectos generales de la anemia infecciosa equina. RSD 11:e51011528508. 10.33448/rsd-v11i5.28508

[CR39] Vieira R, Dias R, Ferreira F, Dias R, Grisi Filho J, Heinemann M (2023) Situação epidemiológica da Anemia Infecciosa Equina no estado do Paraná, Brasil. Sem Ci Agr 44(4):1557–1570

[CR40] Villa-Mancera A, Villegas-Bello L, Campos-García H, Ortega-Vargas S, Cruz-Aviña J, Patricio-Martínez F (2024) Prevalencia del virus de la anemia infecciosa equina en caballos y burros determinada mediante comparación de ELISA y AGID en México. Arq Bras Med Vet Zootec 76:180–186

[CR41] Zhang Z, Guo K, Chu X, Liu M, Du C, Hu Z, Wang X (2024) Desarrollo y evaluación de una tira reactiva para la detección rápida de anticuerpos contra el virus de la anemia infecciosa equina. Appl Microbiol Biotechnol 108(1):85. 10.1007/s00253-023-12980-9 38189948 10.1007/s00253-023-12980-9 PMC10774152

